# Photon Starvation Artifact Reduction by Shift-Variant Processing

**DOI:** 10.1109/access.2022.3142775

**Published:** 2022-01-20

**Authors:** GENGSHENG L. ZENG

**Affiliations:** Department of Computer Science, Utah Valley University, Orem, UT 84058, USA; Department of Radiology and Imaging Sciences, University of Utah, Salt Lake City, UT 84108, USA

**Keywords:** Image processing, image reconstruction, biomedical imaging, computed tomography, filters

## Abstract

The x-ray computed tomography (CT) images with low dose are noisy and may contain photon starvation artifacts. The artifacts are location and direction dependent. Therefore, the common shift-invariant denoising filters do not work well. The state-of-the-art methods to process the low-dose CT images are image reconstruction based; they require the raw projection data. In many situations, the raw CT projections are not accessible. This paper suggests a method to denoise the low-dose CT image using the pseudo projections generated by the application of a forward projector on the low-dose CT image. The feasibility of the proposed method is demonstrated by real clinical data.

## I. INTRODUCTION

The main drive force of using low-dose x-ray computed tomography (CT) is to reduce the patient radiation exposure [1]–[3]. Even though it is not clear whether x-ray radiation exposure plays a role in getting cancers, it is advised to reduce the x-ray exposure to an As-Low-As-Reasonably-Achievable (ALARA) level [4].

An immediate negative effect of using a low dose in CT imaging is that the images become noisy. When the x-ray dose is extremely low, the photon starvation effect can cause severe streaking artifacts in the CT reconstruction [5], [6]. Reference [7] states that “Photon starvation is one source of streak artifact which may occur in CT. It is seen in high attenuation areas, particularly behind metal implants.” In [8] we read: “When too few photons reach detector elements, strong streaks appear through paths of high X-ray attenuation and an image becomes completely useless.”

The conventional denoising methods are based on the shift-invariant assumption. They can be implemented either in the spatial-domain as convolution methods or in the Fourier-domain as multiplication methods. The conventional denoising methods include many classic linear filters, for example, Butterworth filters [9], Hamming filters [10], Hanning filters [11], Gaussian filters [12], moving average filters [13], and autoregressive filters [14]. These linear filters are easy to implement and computationally efficient. They are also shift-invariant.

Shift-invariant filters can also be nonlinear. The nonlinear filters may outperform the linear filters in terms of sharp edge preservation. The median filters [15] and Huber filters [16] are effective denoising filters while maintaining the edges.

Some modern filters are adaptive, and their characteristics depend on the image local patch. Guided filters [17] and bilateral filters [18] are in this category. The transform-based BM3D filter [19] is considered state-of-the-art in image denoising. The BM3D method uses specific nonlocal image modeling by grouping mutually similar 2D image blocks and stacking them together in 3D arrays.

Convolutional neural network (CNN) based methods can be very effective in removing noise from the images provided a large amount of noisy/noiseless image pairs are available to train the neural network [20], [21]. The noisy images should be reconstructed using the projections acquired with similar imaging parameters and similar body anatomy.

This paper presents an effective nonlinear shift-variant procedure that does not need any image pairs to train. This proposed procedure blends the concepts of linear filtering, shift-variant filtering, and tomography. The feasibility and effectiveness of the proposed procedure are illustrated by its application to real clinical data.

## II. METHODS

### A. UNIQUENESS OF THE PROBLEM

The main focus of this paper is the removal of the photon starvation artifacts in low-dose CT. Artifacts are nonstationary noise and normally have some recognizable structures or textures. Photon starvation artifacts appear as bright and dark streaks along the direction where the x-rays have the largest attenuation. For human torso scans, this worse-case direction is the shoulder-to-shoulder direction.

Due to the location dependency of the artifacts, a linear convolutional filter is not effective, which will smooth the entire image but cannot remove the streaking artifacts, as demonstrated in the Results section of this paper.

Shift-invariant nonlinear filters are not effective, either. When the artifacts are severe, the nonlinear filters fail to correctly identify the artifacts to remove and the true edges to keep.

The state-of-the-art methods in reducing the noise/artifacts in low-dose CT images are image reconstruction based [22]–[24]. In other words, the noise control is performed during image reconstruction. Iterative reconstruction algorithms are suitable for noise control image reconstruction. One strategy in an iterative image reconstruction is to assign a weight to each projection. A less-noisy projection is assigned with a larger weight; a noisier projection is assigned with a smaller weight. As a result, the noisier projections have less influence on the reconstructed image. A typical objective function for the iterative image projection contains the following data fidelity term

(1) $$F=\sum_{i=1}^{N}w_{i}{\left(a_{i}^{T}x-p_{i}\right)}^{2},$$

where *x* is the (unknown) image represented as a column vector, *p*_*i*_ is the *i*th projection (i.e., line integral), *w*_*i*_ is the weighting factor for the *i*th measurement, and the inner product $a_{i}^{T}x$ represents the pseudo (simulated) forward projection of the *i*th projection. Image reconstruction is to minimize the objective function *F* in (1) to obtain an estimation of the image *x*.

The objective function (1) only has the data fidelity enforcement. The objective function can contain some Bayesian terms, for example, a total variation (TV) norm of the image *x* [25].

In this paper, we assume that the image *x* is already some-how reconstructed, for example, by the analytical filtered backprojection (FBP) algorithm [26]. The image is noisy and contains photon starvation artifacts. The original measured projections are NOT available anymore.

### B. THE PROPOSED ALGORITHM

The proposed artifact reduction algorithm is introduced as follows.

Step 1. For a given image *x*_*old*_, generate simulated pseudo projections as
(2) $$p_{i}=a_{i}^{T}x_{old}$$
  for all *i*.
Step 2. Select a threshold value *T*.
Step 3. Loop through all projections *p*_*i*_.
  If *p*_*i*_ < *T*, do nothing.
  If *p*_*i*_ ≥ *T*, replace *p*_*i*_ by its filtered version using a one-dimensional moving-average filter along the detector direction.
Step 4. Apply the filtered backprojection (FBP) algorithm to the processed pseudo projections, to obtain the final image *x*_*new*_.

The threshold value *T* is a user-selected parameter, and we used *T* as the 75% of the maximum projection value in our study in this paper.

We now explain what motivates this algorithm. We do not choose any shift-invariant filters, because the artifacts are location and direction dependent. Since the state-of-the-art denoising algorithms are image reconstruction based, we choose an image reconstruction-based algorithm.

Our biggest obstacle is that we do not have an access of the original measurements in the projection domain. We only have a noisy reconstruction *x*_*old*_. The simulated pseudo forward projection $a_{i}^{T}x_{old}$ is not the same as the originally measured projection.

The original projections due to noise are inconsistent. The inconsistency carries the noise information. The inconsistency information is lost in the forward projection $a_{i}^{T}x_{old}$ The objective function *F* in (1) is already at its minimum with the pseudo projections because $p_{i}=a_{i}^{T}x_{old}$ Therefore, the strategy of selecting a set of weights to minimize *F* in (1) does not help.

Realizing that the re-projected pseudo measurements do not carry the same information and do not have the same values as the original raw measurements, our novel strategy of this paper is to use the transmission data noise model to estimate the noise variance in the re-projected pseudo measurements.

When the original x-ray photon counts *I*_0_ pass through an object, according to the Beer-Lambert law [27], the survived photon counts *I* can be estimated as

(3) $$I=I_{0}e^{-p}$$

where *p* is the line integral of the attenuation coefficients along the projection ray passing through the object. This line integral *p* is referred to as the ‘projection’ or ‘post-log measurement’ or simply ‘measurement’ in tomography.

It is assumed that the noise in the survived photon counts *I* follows the Poisson distribution, and its variance is the same as its mean value. Using the expression

(4) $$p=\text{ln}\;\left(I_{0}\right)-\text{ln}\;(I),$$

the variance of *p* can be estimated as

(5) $$\text{var}(p)={\left|\frac{\partial p}{\partial I}\right|}^{2}\text{var}(I)=\frac{1}{I^{2}}\text{mean}(I)=\frac{1}{I}=\frac{e^{p}}{I_{0}},$$

where we approximate mean(*I*) by *I* because we only have one realization of the random variable *I*. The value *I*_0_ in practice is very large (maybe in the range of 10^3^ ~ 10^4^) and can be approximated as a noiseless constant.

From (5), the variance of the random variable *p* is an exponential function of *p*, assuming that *I*_0_ is a constant. The relation (5) tells us that the line integral *p* is very noisy if *p* is large. This is the reason of using a threshold *T* in the proposed algorithm to determine if the random variables *p*_*i*_ are too noisy and need to be filtered.

When the noise variance is too large, a lowpass filter is applied to smooth out the noise. Our experience shows that this lowpass filter is very ‘forgiving’ in the sense that we do not need to worry about over-smoothing. The choice of using a moving-average filter is based on the consideration that the moving-average filter is the easiest filter to design and to implement. The order (i.e., the size) of the moving-average filter can be chosen to be large enough to remove the artifacts.

The FBP algorithm is selected to reconstruct the final image, because it is fast and easy to implement.

### C. CLINICAL DATA

This paper uses a clinical CT scanner to acquire data. The scanner was Aquilion ONE^™^, made by Toshiba America Medical Systems, Tustin, CA, USA. The data acquisition of a cadaver torso was performed at Leiden University Medical Center. The cone-beam data acquisition had 320 rows. Each row was 0.5 mm tall and had 896 channels (i.e., 896 detectors). The fan angle was 49.2°. The number of views was 1200 over 360°. Two current settings were used for the x-ray tube: 60 mAs for the low-dose acquisition and 500 mAs for the regular-dose acquisition. The images were reconstructed with the FBP algorithm. These imaging system setup parameters are not necessary when our proposed algorithm is applied.

### D. IMAGE EVALUATION

The most common way to determine the effectiveness of artifact removal algorithms is by visual inspection or human observer studies. A quantitative evaluation metric adopted in this paper is the Sum Square Difference (SSD), defined as

(6) $$SSD=\frac{\sum_{i,j}{\left[X_{\text{gold}}(i,j)-X(i,j)\right]}^{2}}{\sqrt{\sum_{i,j}{\left[X_{\text{gold}}(i,j)\right]}^{2}\sum_{i,j}{\left[X(i,j)\right]}^{2}}},$$

where *X*_*gold*_ is the gold standard image, which is the FBP reconstruct from the regular-dose projections, and *X* is another image to compare with. The SSD essentially is the normalized distance between two images *X*_*gold*_ and *X*.

A second quantitative evaluation method adopted in this paper uses the noise power spectrum image, which is defined as the magnitude image of the two-dimensional (2D) Fourier transform of the difference image of *X*_*gold*_ − *X*. If these two images exactly match, this noise power spectrum image is a constant zero. This noise power spectrum image can tell if the two images are the same and the frequency components of the differences.

A third quantitative evaluation method in this paper is the modulation transfer function (MTF), which is defined as

(7) $$MTF=\frac{\left|FT\left(X_{\text{output}}\right)\right|+\epsilon }{\left|FT\left(X_{\text{input}}\right)\right|+\epsilon },$$

where *FT* represents 2D Fourier transform of an image, *ε* is a small positive number to prevent the denominator being zero, *X_input_* is the input image, and *X_output_* is the output image. In this paper, *ε* is selected as 0.1. The output image *X*_*output*_ is the processed image, and the input image *X*_*input*_ is the unprocessed image. In this paper, the input image can be the regular-dose FBP reconstruction or the low-dose FBP reconstruction. The MTF defined in (7) is an image. However, the MTF curves are easier to understand. In this paper, from the MTF image we extract 3 MTF curves, the 0° curve, 45° curve, and the 90° curve, according to the procedure descripted in [28]. The MTF images are first transformed in the polar coordinates. The 0° curve is obtained by summing the MTF images from −16° to 16°. The 45° curve is obtained by summing the MTF images from 45°−16° to 45°+16°. The 90° curve is obtained by summing the MTF images from 90°−16° to 90°+16°. The purpose of summation is to reduce noise.

**FIGURE 1.**
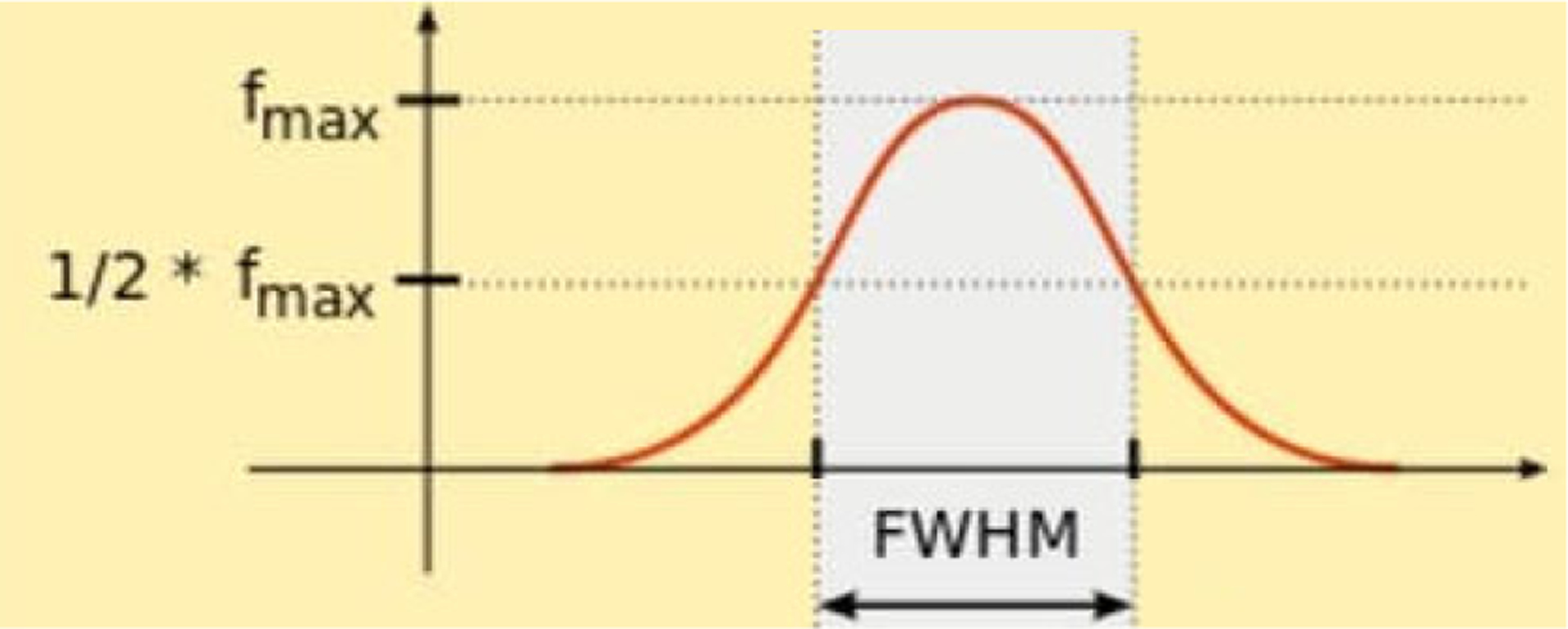
The definition of the full width at half maximum for a pulse function.

A fourth evaluation method is the line profile evaluation to compare the spatial resolution in the image domain, while the second and third evaluation methods are in the frequency domain. A direct result from the line profiles is the full-width-at-half-maximum (FWHM) value, as illustrated in Fig. 1.

## III. RESULTS

**FIGURE 2.**
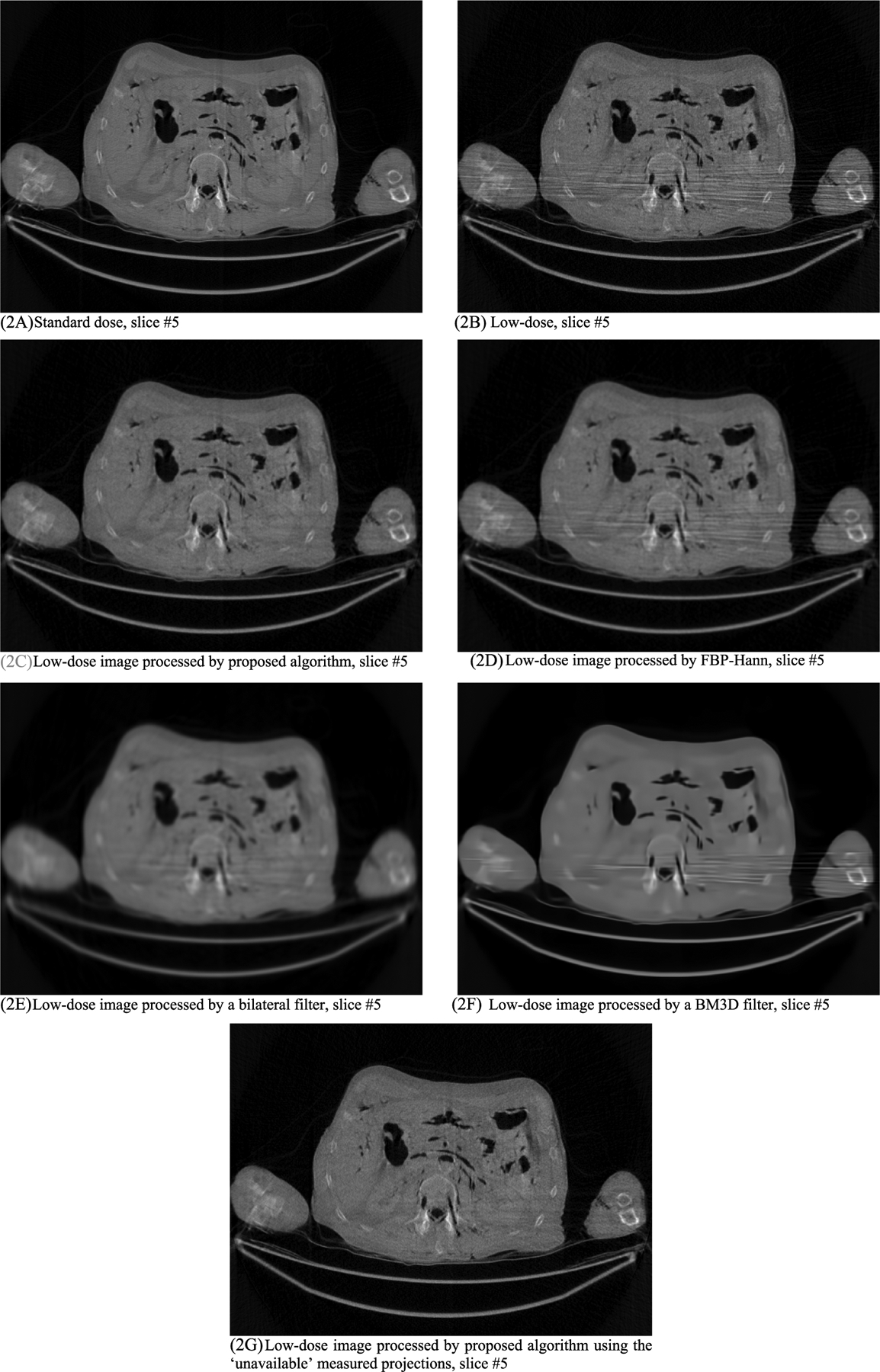
Processed and unprocessed images for slice #5. The standard-dose image in (2A) is the gold standard. The image with the proposed method (2C) gives the best result among all images using the low-dose raw image (2B). The image in (2G) uses the ‘unavailable’ measured data.

**FIGURE 3.**
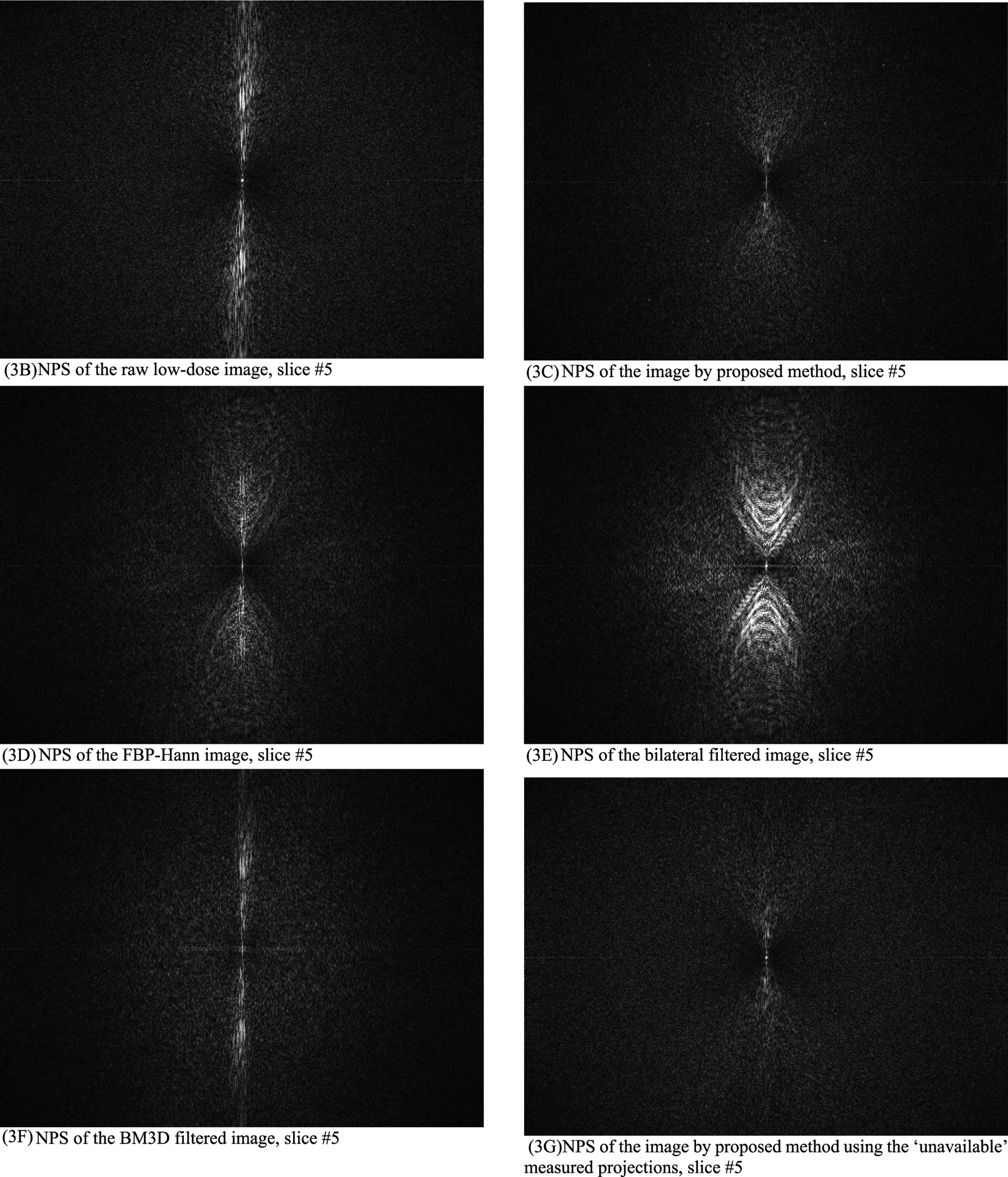
The noise power spectrum images of the processed images for slice #5. The proposed method (3C) has the fewest concentrated bright dots along the central vertical axis. (3A) is a constant 0 (not shown).

**FIGURE 4.**
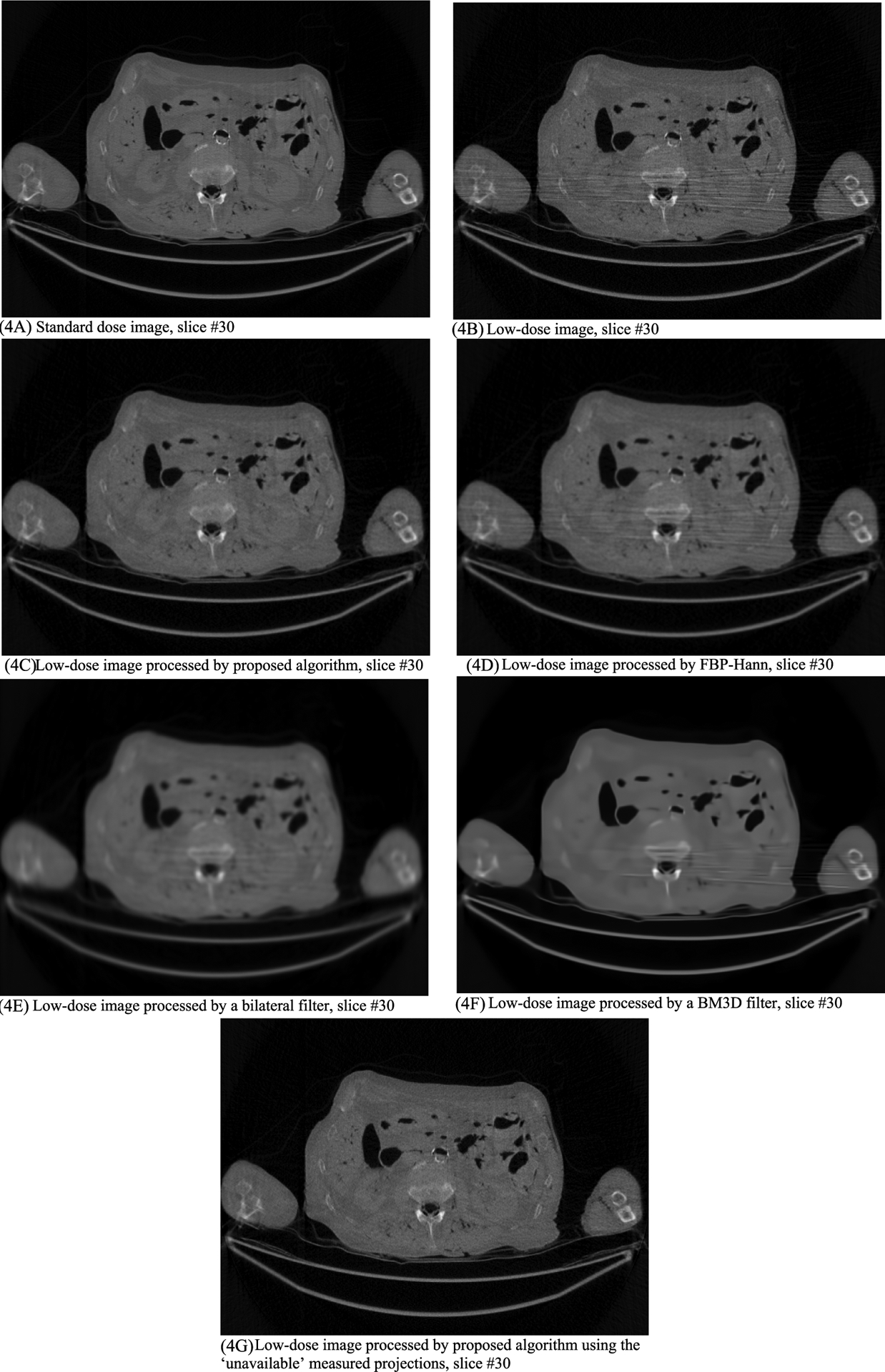
Processed and unprocessed images for slice #30. The standard-dose image in (4A) is the gold standard. The image with the proposed method (4C) gives the best result among all images using the low-dose raw image (4B).

**FIGURE 5.**
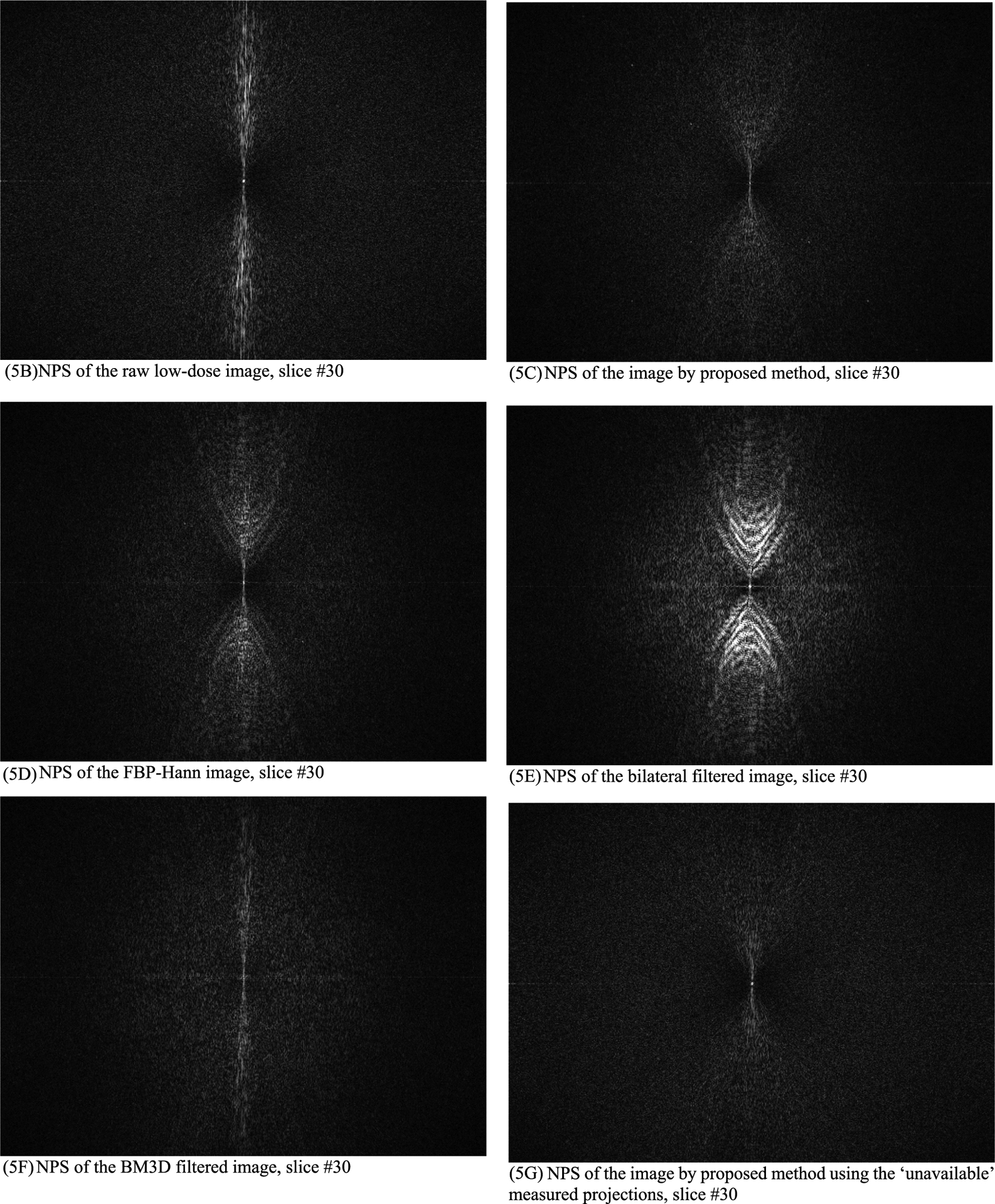
The noise power spectrum images of the processed images for slice #30. The proposed method (5C) has the fewest concentrated bright dots along the central vertical axis. (5A) is a constant 0 (not shown).

**FIGURE 6.**
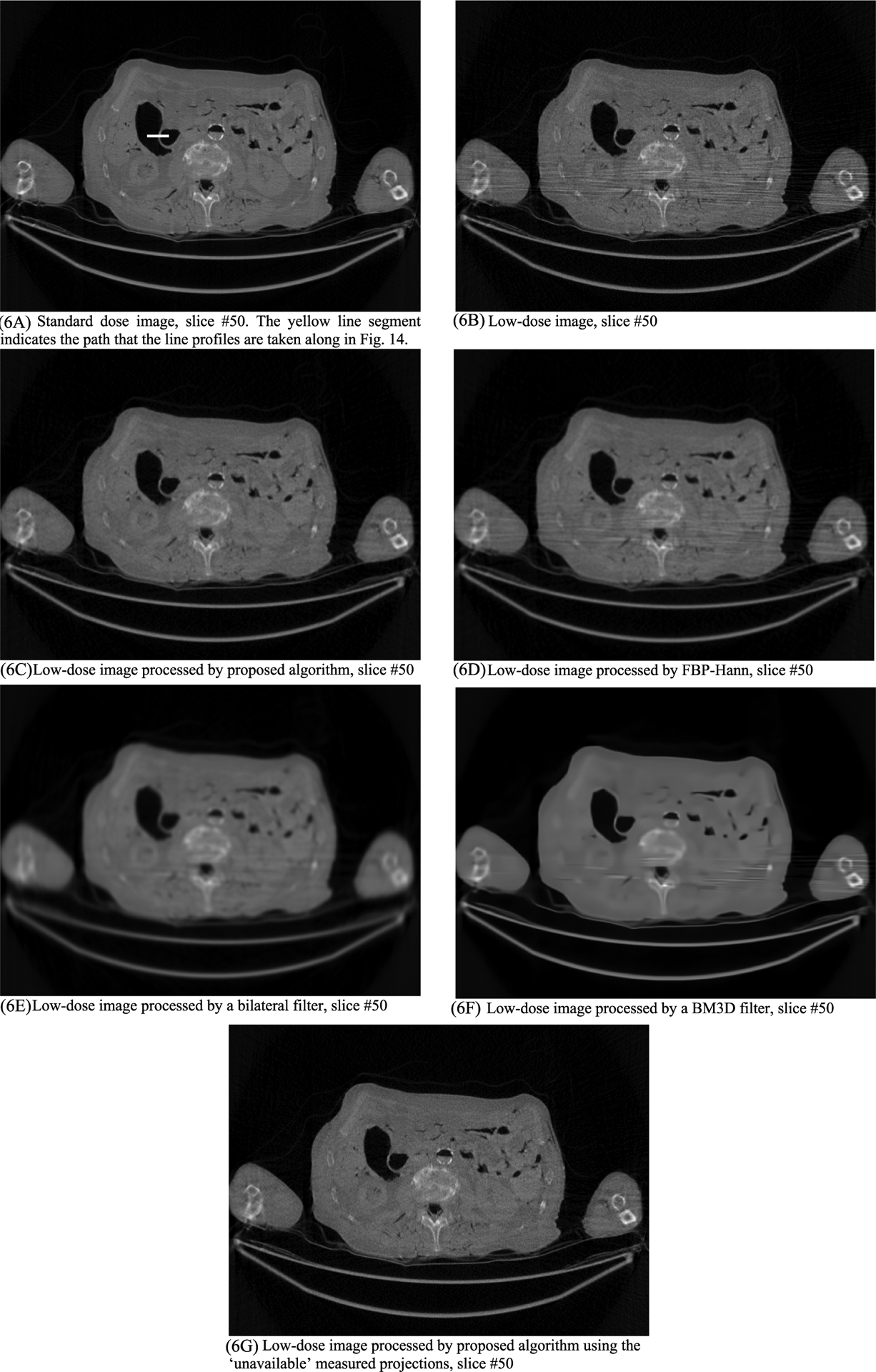
Processed and unprocessed images for slice #50. The standard-dose image in (6A) is the gold standard. The image with the proposed method (6C) gives the best result among all images using the low-dose raw image (6B).

**FIGURE 7.**
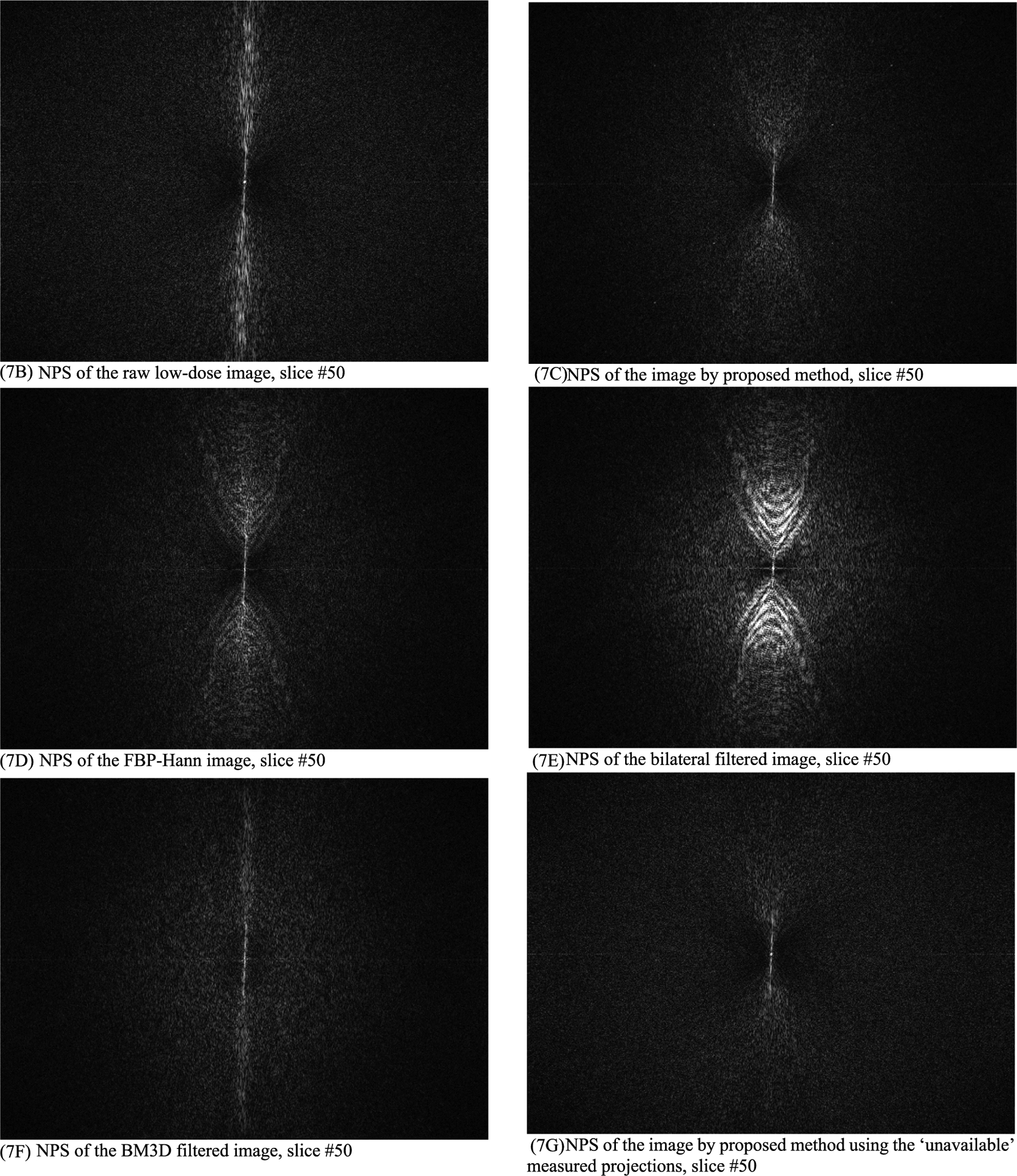
The noise power spectrum images of the processed images for slice #50. The proposed method (7C) has the fewest concentrated bright dots along the central vertical axis. (7A) is a constant 0 (not shown).

**FIGURE 8.**
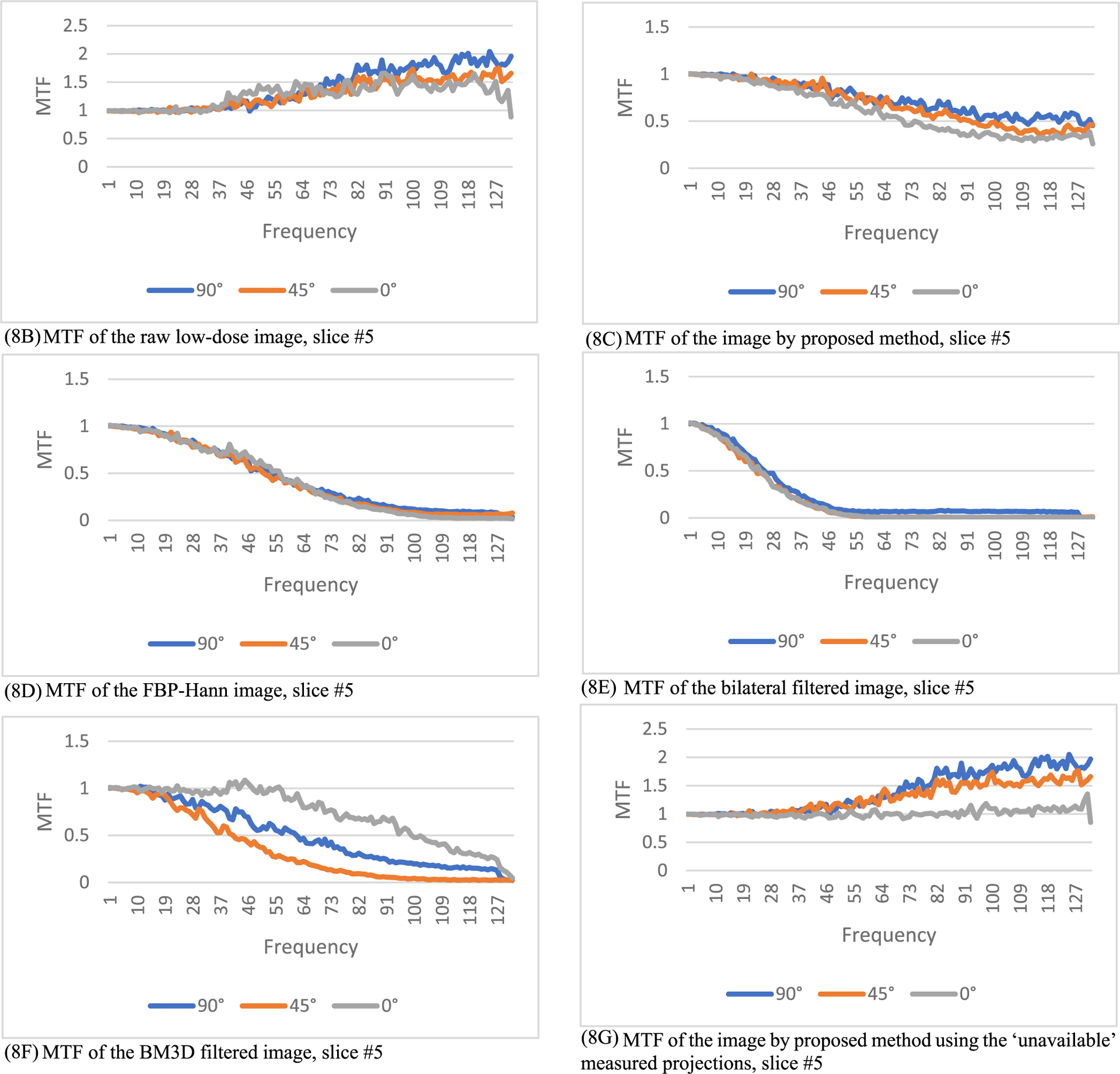
The modulation transfer function images of the processed images for slice #5. The proposed method (8C) has the smallest high-frequency noise structures. (8A) is a constant 0 (not shown).

**FIGURE 9.**
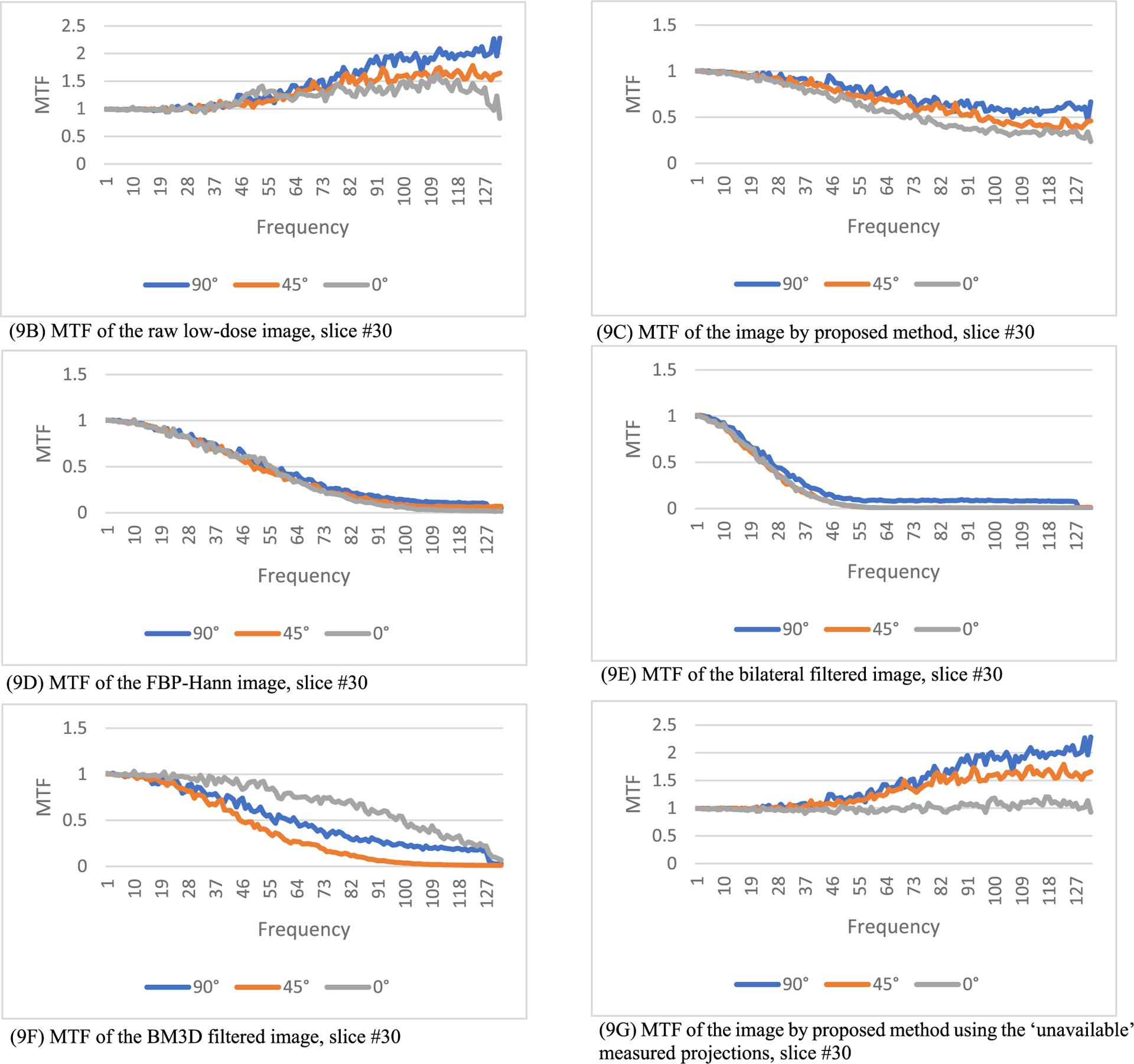
The modulation transfer function images of the processed images for slice #30. The proposed method (9B) has the smallest high-frequency noise structures. (9A) is a constant 0 (not shown).

**FIGURE 10.**
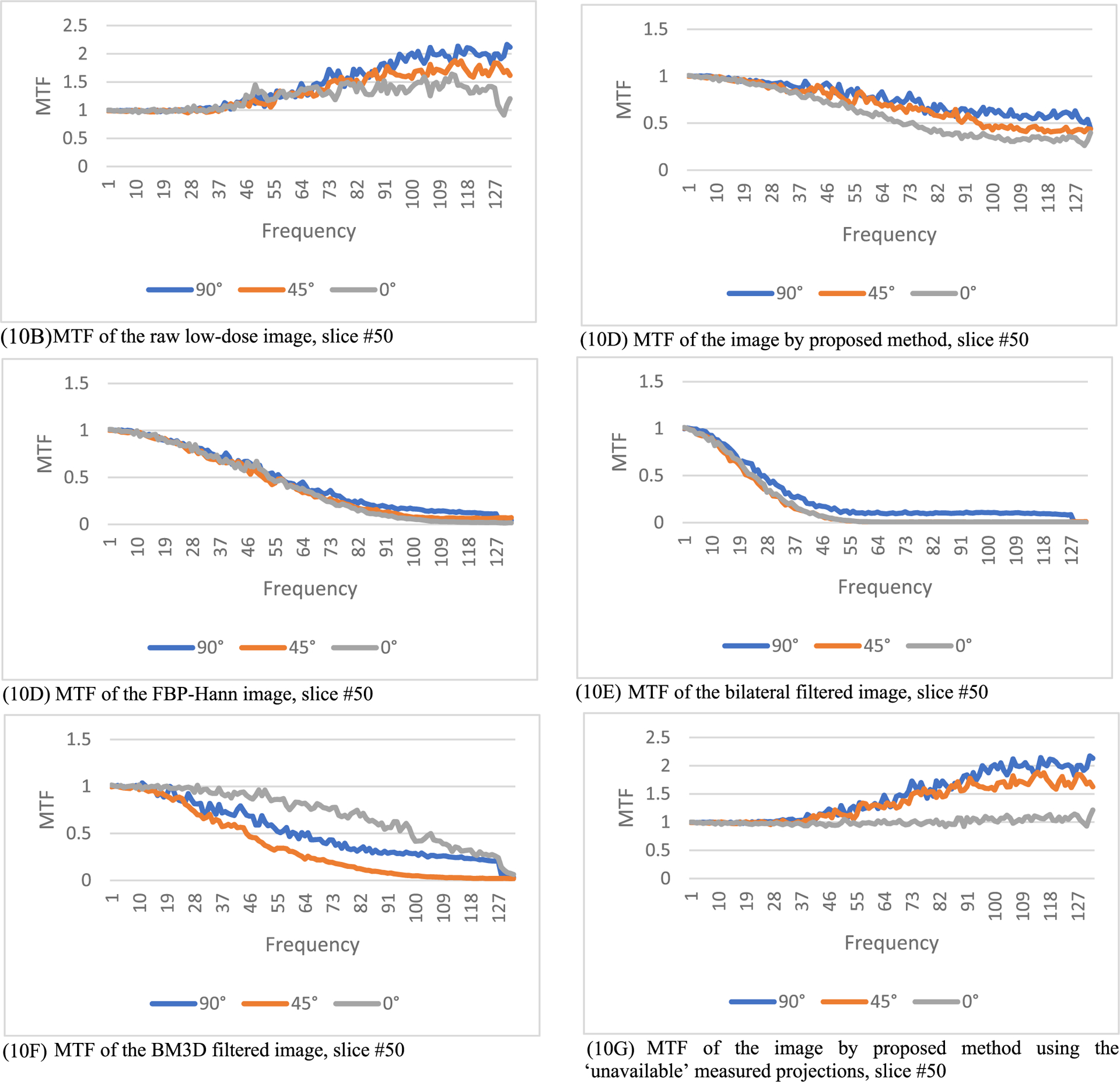
The modulation transfer function images of the processed images for slice #50. The proposed method (10C) has the smallest high-frequency noise structures. (10A) is a constant 0 (not shown).

**FIGURE 11.**
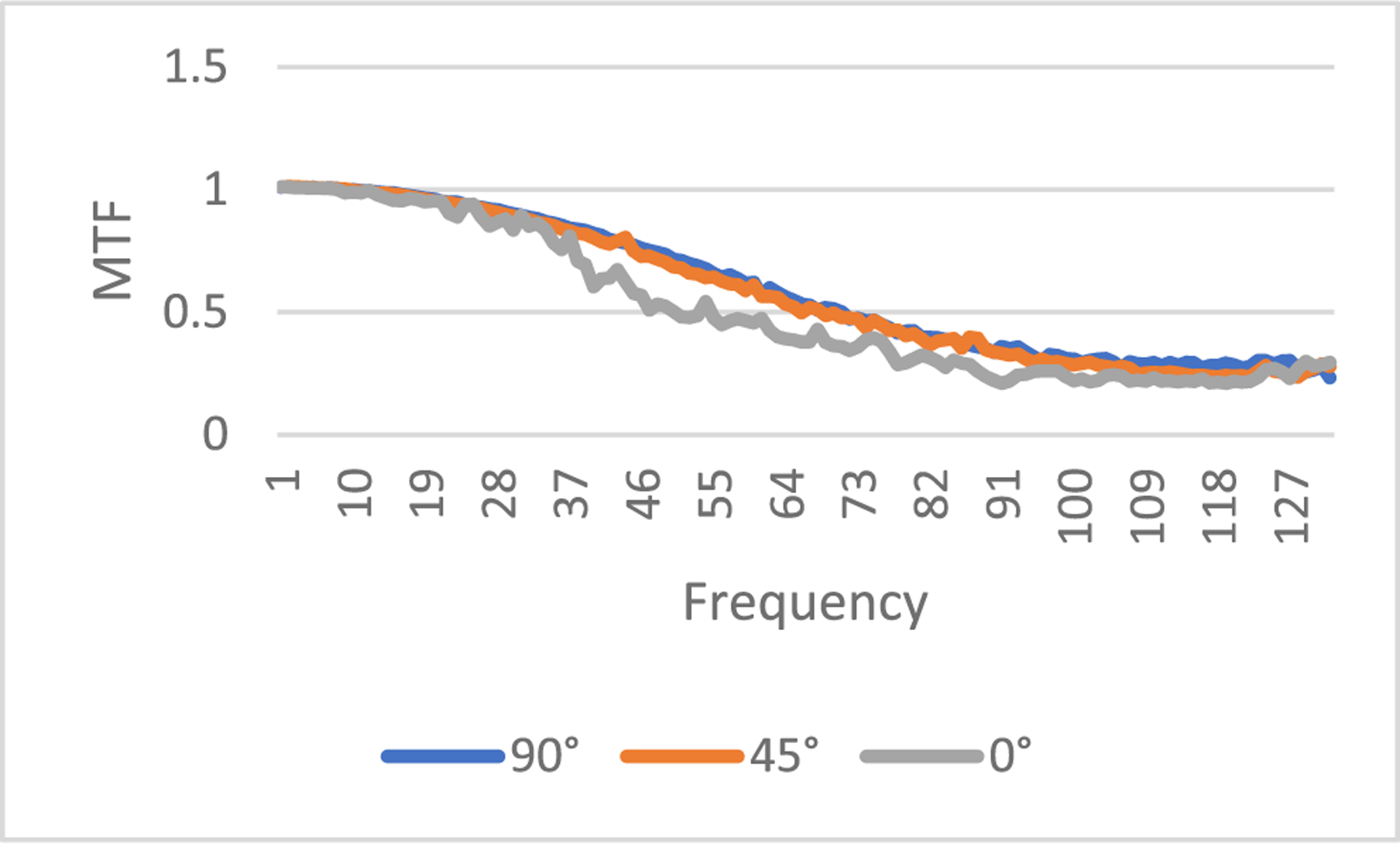
MTF from the low-dose FBP image to the image by the proposed method, slice #5.

**FIGURE 12.**
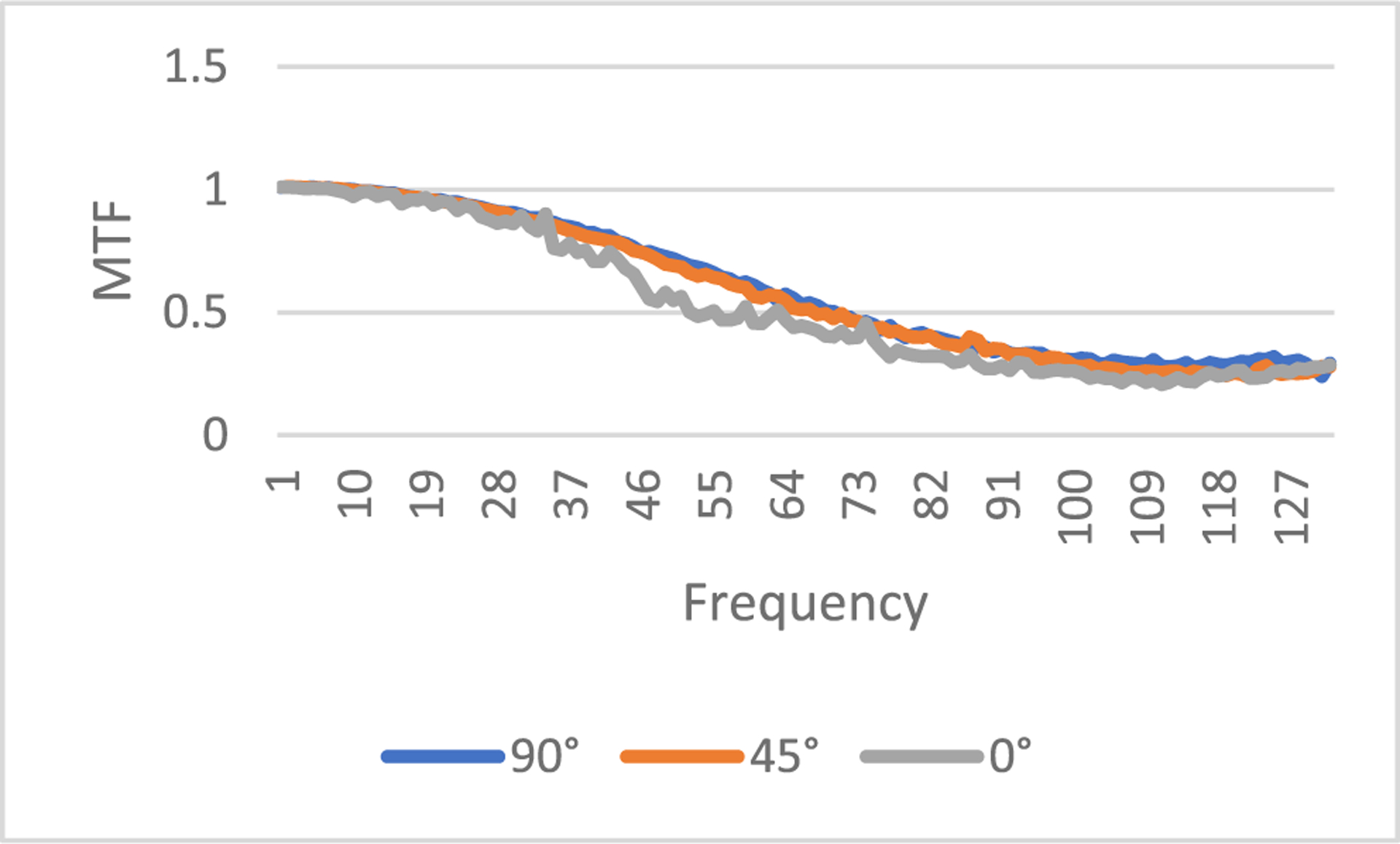
MTF from the low-dose FBP image to the image by the proposed method, slice #30.

**FIGURE 13.**
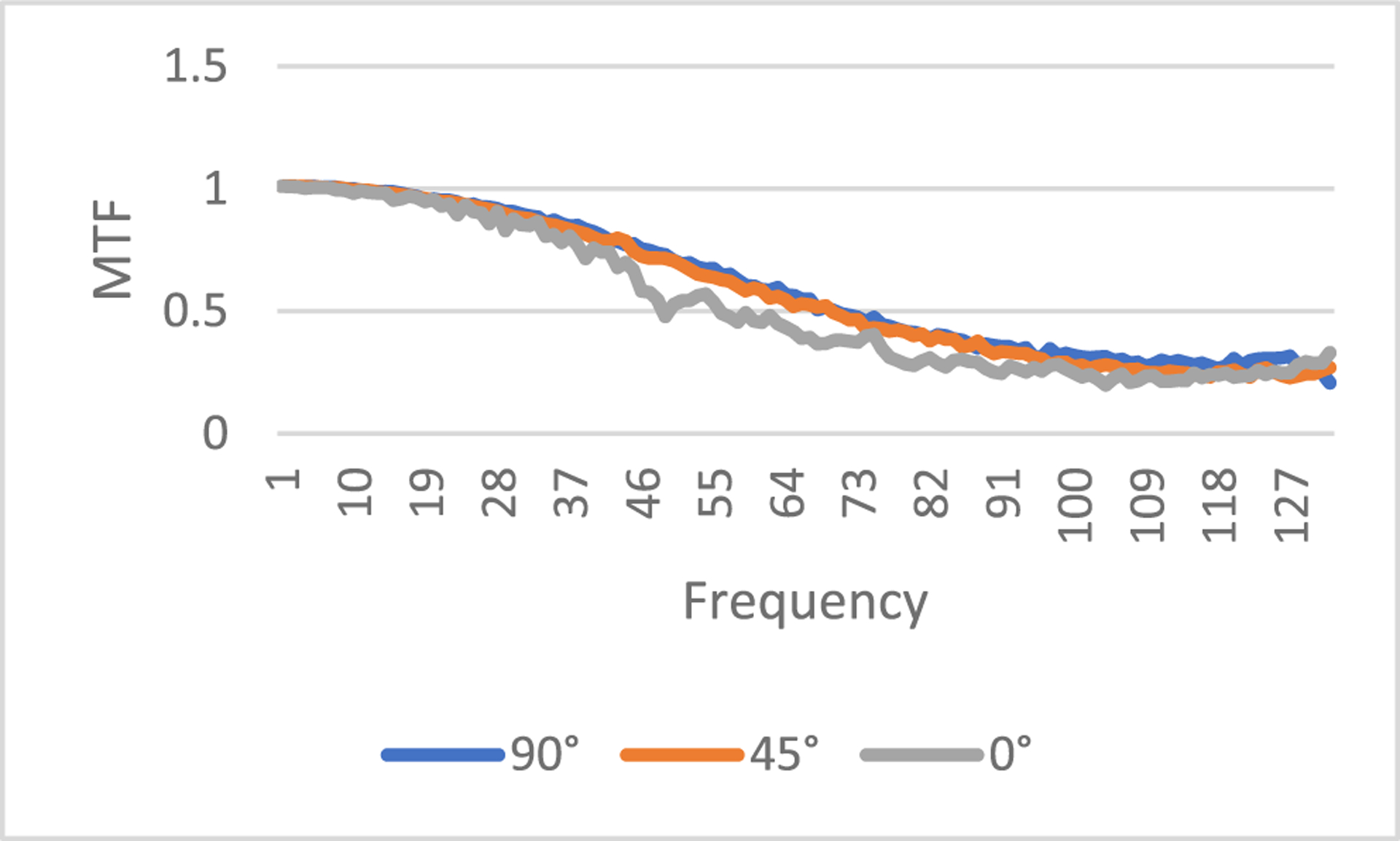
MTF from the low-dose FBP image to of the image by the proposed method, slice #50.

**FIGURE 14.**
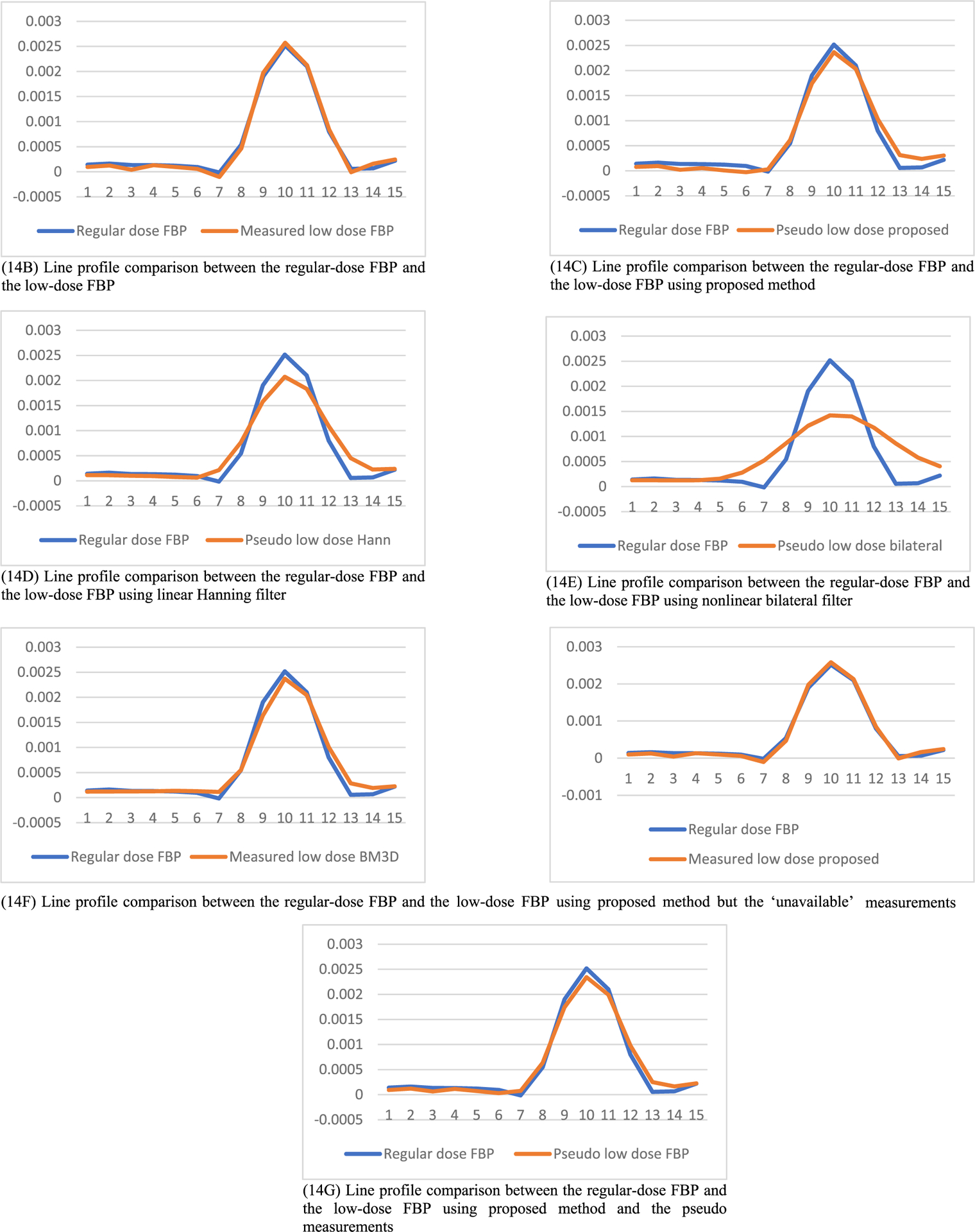
Line profiles along the yellow horizontal line segment indicated in Fig. 6A for slice #50. (14A) comparing the blue curve to itself (not shown).

In this section, the methods are labeled with A – G. We point out that methods A and F use the ‘unavailable’ projections. In Figs. 1–14, the following labels are used for the images: (A) the gold standard image FBP reconstructed from the regular-dose x-ray projections; (B) the raw FBP reconstruction image reconstructed from the measured low-dose x-ray data; (C) the processed image using the proposed algorithm in the paper using the pseudo data; (D) the image is FBP reconstructed with a linear Hanning filter applied to the pseudo data; (E) the image is FBP reconstructed with a nonlinear bilateral filter applied to the pseudo data; (F) the image is post processing result of image from (B) with a BM3D filter in the image domain; (G) almost the same as (C) except that the ‘unavailable’ low-dose x-ray data is used instead of the pseudo data.

Results of three slices are shown. The images from slices #5, #30, and #50 are displayed in Figs. 2, 4, and 6, respectively. Their associated noise power spectrum images are displayed in Figs. 3, 5, and 7, respectively.

The numerical results of the Sum Square Difference (SSD) values are listed in Tables 1, 2, and 3, respectively, for slices #5, #30, and #50. The SSD is a non-negative quantity, the smaller value the better. The ideal SSD value is 0. In all these cases, the proposed method gives the smallest SSD values, indicating the best performance. It is interesting to notice that the proposed method performs better with the pseudo forward projections than with the ‘unavailable’ original low-dose projections. This phenomenon could be caused by the lowpass filtering effect of the forward projecting procedure, which reduces some noise.

The ideal noise power spectrum is a constant 0 (i.e., black color in the image). The noise power spectrum images in Figs. 3, 5, and 7 all have some bright dots in the spectrum images scatter all over. Those scattered bright dots represent noise. The artifact related bright dots are *concentrated along the central vertical axis*. The noise power spectrum images for the proposed method (see C and G) contain the fewest concentrated bright dots along the central vertical axis.

The modulation transfer function (MTF) represents the input-output relationship in the frequency domain. If the output is the same as the input, the MTF is a constant 1. If the input is the regular-dose image, the MTF results are shown in Figs. 8, 9 and 10, respectively, for slices #5, #30, and #50. The 3 MTF curves for the proposed method (see C and G) are very close to each other for the 3 directions and stay away from 0.

If the input is the ‘unavailable’ low-dose projections and the output is processed projections by the proposed method, the MTF images are shown in Figs. 11, 12, and 13, respectively, for slices #5, #30, and #50.

The line profiles shown in Fig. 14 are along the yellow horizontal line segment indicated in Fig. 6A for slice #50, where the yellow line segment is the path that the line profiles are taken. The FWHM values can be readily obtained by the line profiles shown in Fig. 14. The FWHM values are listed in Table 4. The proposed method provides the best resolution among the processing methods. The important fact is that the results from linear Hanning filter, the nonlinear bilateral filter and the BM3D filter show very severe artifacts and loss of small details.

The MTF results in Figs. (8B), (9B), and (10B) indicate some values above 1 at high frequencies. They are caused by the high frequency noise and artifacts.

The MTF results in Figs. (8C), (9C), and (10C) indicate some values below 1 at high frequencies. They are caused by the resolution degradation generated by the pseudo forward projections. This effect can also be observed in Table 4. When the bare-bone FBP is applied to the measured low-dose data, the HWHM is 2.34 and when applied to the pseudo data, the HWHM is 2.98. The pseudo data resolution loss is noticeable. We should always use the original measured data whenever it is available.

**FIGURE 15.**
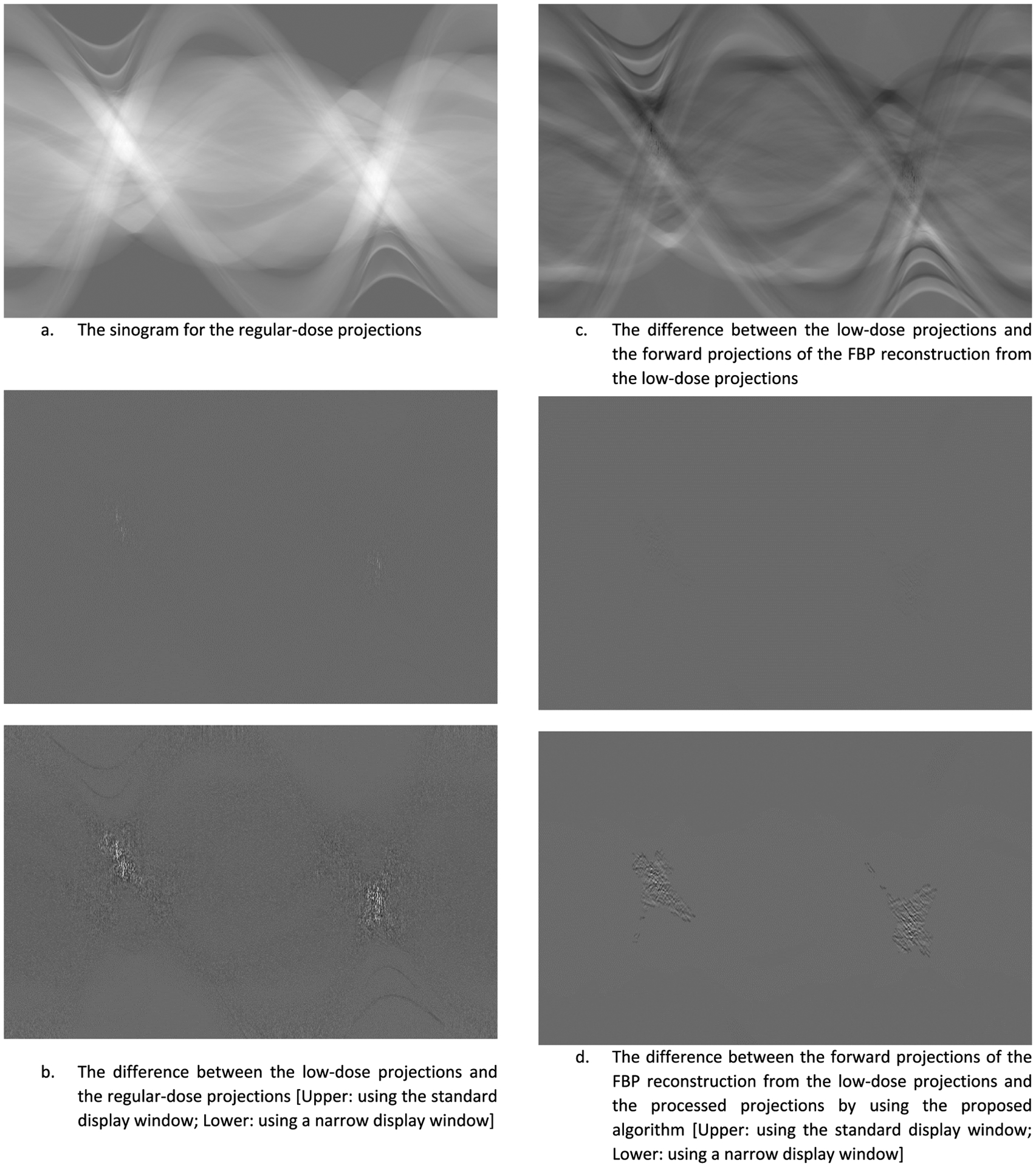
Sinogram domain images of slice #50.

For slice #50, the projection-domain images (also known as the sinograms) are displayed in Fig. 15. The images are (a) the ‘unavailable’ regular-dose projections, (b) the difference between the raw ‘unavailable’ low-dose projections and the ‘unavailable’ regular-dose projections, (c) the difference between the pseudo forward projections from the low-dose FBP reconstruction and the ‘unavailable’ low-dose projections, and (d) the difference between the processed version of the pseudo forward projections from the low-dose FBP reconstruction and the unprocessed version, respectively. It is observed from Fig. 15d that the proposed method only alters a very small portion of the projections.

All images in this paper are displayed with the linear gray scale. The largest image value is white; the smallest image value is black. For the CT images, the display window is from 0 to the maximum image value of the regular-dose image. For the sinogram images, the display window is [−V, V], where V is maximum value of the regular-dose sinogram. This standard window [−V, V] is too wide when displaying the sinogram differences. A narrower display window is also used when the sinogram image shows almost a constant. For the noise spectrum images, the display window is [0, 0.5].

The proposed method is able to remove the shoulder-to-shoulder bright-and-dark streaking artifacts while keep the high contracts in the artifact-free regions.

The kernel in the moving-average filter (See Step 2 in the algorithm) in the proposed algorithm has 13 elements.

The proposed algorithm is effective in reducing the streaking artifacts and keeping the image resolution. As a comparison, the images produced by a linear Hanning filter, a nonlinear bilateral filter, or a BM3D filter are unable to to keep small details while the streaking artifacts are still severe.

The proposed algorithm was implemented in Matlab® on a Thinkpad laptop computer with Windows 10. The processor was Intel® Core^™^ i7-10510U CPU @ 1.80GHz. The processing time was 0.56 seconds.

## IV. DISCUSSION

When a noisy reconstructed image is available while the original projection measurements are no longer available, the pseudo re-projected line integrals are not helpful to reduce noise if a conventional iterative image reconstruction algorithm is to be used. The conventional iterative image reconstruction algorithms work in the principle of reducing the data fidelity term as presented in (1), where the entire summation on the right-hand side is referred to as ONE fidelity term. By using the pseudo re-projected line integral data, this data fidelity term is already at its minimum value, which is zero.

One way of denoising is to stop the iterations early. This approach is equivalent to lowpass filtering, which is shift invariant. As we demonstrated in the Results section, shift-invariant denoising smooths the images but still cannot reduce the streaking artifacts.

A filter is referred to as shift-*in*variant if the filter operation is the same everywhere. In our proposed filter, the filter operation is only applied to a small amount of selected pseudo projections. Therefore, our proposed filter is shift variant.

A linear filter must satisfy the linear scaling property that if the image *X* results in a filtered version *Y* after filtering, then a scaled version of *X*, *αX*, should result in *αY* after filtering, where is a real number. Our proposed filter is associated with a threshold value *T*; the scaling factor affects whether the data value is greater or less than the threshold value *T*. Therefore, our proposed filter is nonlinear.

Our proposed algorithm is NOT an iterative image reconstruction algorithm; it is an analytic FBP algorithm with a nonlinear pre-filter. In the FBP algorithm, a ramp filter (which is a high-pass filter) must be used to cancel the bachprojection blurring. The purpose the low-pass filter is to reduce the noise in the image. The application of a low-pass filter is optional in FBP, only when image denoising is necessary. The main goal of this paper is photon-starvation artifact reduction, we do not apply a linear low-pass filter in the FBP algorithm. In the proposed algorithm, there is a threshold value *T*; any pseudo projection data value that is less than this threshold value is not affected. The majority of the pseudo projections are less than this threshold. Thus, the image resolution degradation is kept to its minimum.

Fig. 15b is the low-dose data minus the regular-dose data; it reveals that in the heavily attenuated regions, the low-dose measurements mainly have smaller values than the regular-dose measurements. However, at some scattered points, the low-dose measurements have larger values than the regular-dose measurements. Therefore, we do not have a systematic way to convert the low-dose data into regular-dose data due to complicated beam hardening effects.

Fig. 15c shows that there are significant differences between the ‘unavailable’ measured data and the pseudo re-projected data.

Fig. 15d shows that in the most part of the sinogram space, the pseudo re-projection data and the processed data by the proposed algorithm are the same. The proposed algorithm only alters the measurement values in the heavily attenuated regions.

**FIGURE 16.**
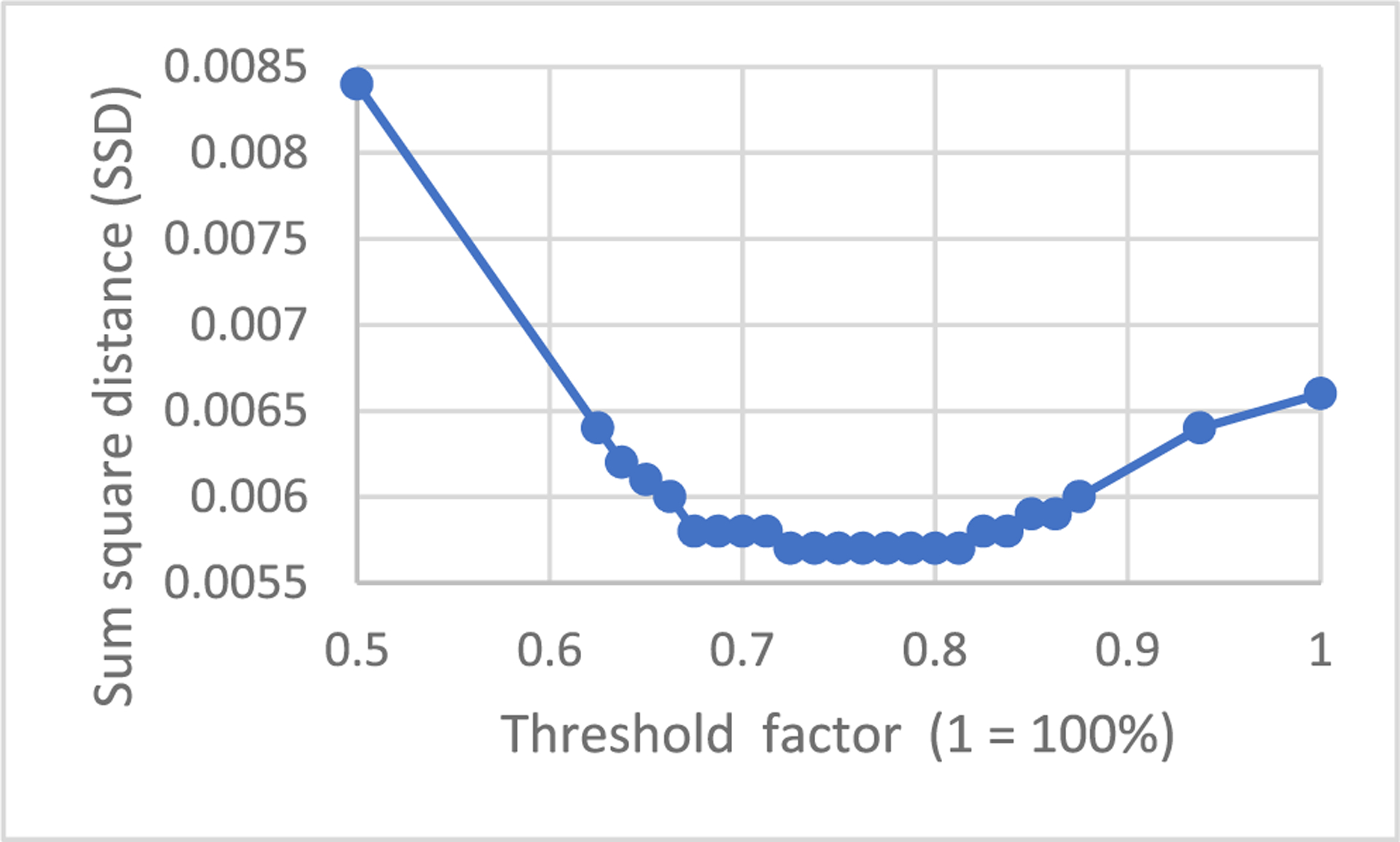
A sensitivity study of the SSD with respect to the threshold value *T* for slice #50.

The proposed algorithm contains a user-determined hyper parameter *T*. This hyper parameter *T* is determined by trial and error. In fact, parameter *T* is not very sensitive. As shown in Fig. 16, the SSD vs *T* curve has a flat valley, which means that a wide range of the parameter *T* can give the optimal solution.

In this paper, the geometry of the original imaging setup is assumed to be unknown. The forward pseudo projection setup can be different and independent from the original setup. The original number of angles in data acquisition was 1200 over 36°. If we use the number of angles in the pseudo projections as 2400 (instead of 1200) over 360°, our proposed algorithm gives almost the same result as that when we use the number of angles in the pseudo projections as 1200 over 360°.

Three noise-reducing filters have been used to compare with the proposed shift variant filter in the task of photon starvation artifact reduction. Those three noise-reducing filters do not perform well for this task. If the filters are adjusted to remove the artifacts, many image details are removed as the price to pay. The message of our paper is that the noise reduction task is different from the artifact reduction task. For artifact reduction, where to filter (or equivalently, where not to filter) is far more important than what filter to use. Once the region to be filtered is identified, many filters are effective as long as the filters have enough smoothing power. We choose the simplest linear moving-average filter with a large enough kernel size. Other noise reduction filters such as bilateral and BM3D filters will work just fine when applied only in the specified region. The critical point is that we do not apply the lowpass filter to the entire image or the entire sinogram.

## V. CONCLUSION

We have developed an effective method to reduce the photon starvation streaking artifacts in low-dose x-ray CT images. The proposed method is shift-variant; it only applies lowpass filtration for some pre-determined measurement values in the sinogram domain.

We assume that the raw, low-dose measurements are not available, and the noisy reconstruction is available. A set of pseudo re-projections are generated from noisy reconstruction. A threshold value *T* is selected by the user using a trial-and-error method. The pre-determined measurements are selected if the pseudo measurement value is greater than *T*. The pre-selected pseudo measurements are filtered in the sinogram domain by a simple moving-average lowpass filter along the detector direction. The FBP algorithm is performed to generate a final image using the selectively filtered pseudo measurements.

The effectiveness of the proposed method has been demonstrated by real clinical data. The proposed method is not restricted to CT images. Other potential applications include processing images that are contaminated with textures along some particular directions.

## Figures and Tables

**TABLE 1.** Sum square distance (SSD) between the processed low-dose image and the regular-dose image (Slice #5).

Image (Methods: B – F)	SSD value
B. Raw low-dose image	0.0119
C. Processed by proposed method	0.0060
D. Processed by FBP-Hann	0.0081
E. Filtered by a bilateral filter	0.0192
F. Processed by a BM3D filter	0.0072
G. Processed by proposed method but using the ‘unavailable’ low-dose measurements	0.0090

**TABLE 2.** Sum square distance (SSD) between the processed low-dose image and the regular-dose image (Slice #30).

Image (Methods: B – F)	SSD value
B. Raw low-dose image	0.0110
C. Processed by proposed method	0.0057
D. Processed by FBP-Hann	0.0077
E. Filtered by a bilateral filter	0.0189
F. Processed by a BM3D filter	0.0063
G. Processed by proposed method but using the ‘unavailable’ low-dose measurements	0.0088

**TABLE 3.** Sum square distance (SSD) between the processed low-dose image and the regular-dose image (Slice #50).

Image (Methods: B – G)	SSD value
B. Raw low-dose image	0.0113
C. Processed by proposed method	0.0058
D. Processed by FBP-Hann	0.0076
E. Filtered by a bilateral filter	0.0189
F. Processed by a BM3D filter	0.0066
G. Processed by proposed method but using the ‘unavailable’ low-dose measurements	0.0090

**TABLE 4.** Full width at half maximum value comparison.

Method	FWHM (pixels)	Severe Artifacts?
A. Regular-dose FBP	2.54	No
B. Low-dose FBP, using the ‘unavailable’ low-dose measurements	2.34	yes
C. Low-dose FBP using proposed method	2.96	No
D. Low-dose FBP using linear Hann filter	3.76	Yes
E. Low-dose FBP using nonlinear bilateral filter	7.00	Yes
F. Measured Low-dose using BM3D filter	2.55	Yes
G. Low-dose FBP using proposed method, but using the ‘unavailable’ low-dose measurements	2.34	No
H. Low-dose FBP, using the pseudo data	2.98	No
